# V-doped KNbO_3_ perovskite for enhanced photocatalytic hydrogen production from first-principles calculations

**DOI:** 10.1039/d5ra08476h

**Published:** 2026-01-05

**Authors:** Aktab Quadir Sreshtho, Sourav Chandra Das, Tanvir Aftab Talal, Md Hasnain, Joy Biswas, Akdas Quadir Shwapno, Riddi Barua, Moussab Harb

**Affiliations:** a University of Dhaka Faculty of Engineering and Technology Dhaka Bangladesh mechasnain@gmail.com; b Department of Physics, Faculty of Science, King Abdulaziz University Jeddah 21589 Saudi Arabia mharb@kau.edu.sa

## Abstract

Photocatalytic water splitting represents a promising route for sustainable hydrogen production using solar energy. In this study, Density Functional Theory (DFT) is applied to investigate the influence of vanadium (V) doping on the electronic and optical properties of cubic KNbO_3_ (KNO). Simulations on KNb_1−*x*_V_*x*_O_3_(KNVO), with *x* = 0.11, were performed using the CASTEP module under both GGA-PBE and mGGA-RSCAN functionals to reach better computational reliability. The mGGA-RSCAN functional exhibits better accuracy in reproducing the experimental bandgap values. 11.11% V substitution at the Nb site significantly reduces the bandgap from 1.82 eV to 1.53 eV (PBE) and from 2.30 eV to 1.86 eV (RSCAN), enhancing visible-light absorption. Optical and conductivity analyses reveal improved absorption and charge transport properties in V-doped systems. XRD analysis confirms the cubic structural stability. The valence and conduction band edge potentials obtained from Mulliken electronegativity satisfy the redox potential criteria for water splitting. These findings suggest V-doped KNbO_3_ as a viable photocatalyst for visible-light-driven hydrogen production.

## Abstract 2

Photocatalytic water splitting represents a promising route for sustainable hydrogen production using solar energy.

## Graphical abstract

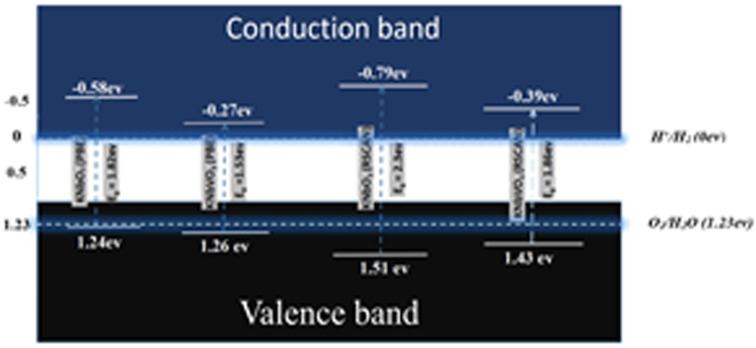

## 1 Introduction

Hydrogen emerges as a highly promising energy carrier due to its high energy density and effective storage properties, which address the intermittency issues of renewable energy sources such as solar and wind. Unlike electricity, which faces transmission losses and stringent voltage requirements,^1^ hydrogen can be transported and stored efficiently using existing chemical fuel infrastructure that enhances its practicality. However, current hydrogen production methods from fossil fuels are economically challenging due to high energy demands and significant CO_2_ emissions.^2^ Therefore, the transition to renewable hydrogen production methods, such as photocatalytic water splitting, becomes imperative.^3,4^ This technique, based on the foundational research by Honda and Fujishima in the 1970s using titanium dioxide (TiO_2_) and platinum (Pt) electrodes, effectively converts solar energy into chemical energy by absorbing solar photons.^5^

Hydrogen production from water splitting using solar energy offers a promising solution to future energy crises, since hydrogen's heat of combustion is significantly higher than that of ethanol and methanol.^6^ In photoelectrochemical (PEC) water splitting, semiconductor materials are used to convert solar energy directly into chemical energy in the form of hydrogen.^7^ The semiconductor is immersed in a water-based electrolyte, where sunlight energizes the water-splitting process. For effective operation, the semiconductor must have a band gap greater than 1.23 eV to enable overall water decomposition.^7^ Materials like TiO_2_, ZnS, and CdS are commonly used in photocatalytic water splitting due to their ability to generate electron–hole pairs under light irradiation.^7^

However, issues like photo-corrosion and low efficiency under visible light limit their use. Various semiconductor materials, including potassium niobate (KNbO_3_ or KNO), have been studied for this purpose.^8–10^ KNbO_3_ with an indirect band gap experimentally measured between 3.2 and 3.3 eV (strongly underestimated in GGA-PBE calculations as ∼1.4–2.0 eV), exhibits phase-dependent photocatalytic activity where the cubic phase outperforms orthorhombic and tetragonal forms due to its favorable symmetry and electronic structure.^11^ Doping is a crucial method for improving KNbO_3_ photocatalytic performance. Adding impurities to the KNO crystal lattice can alter its electrical structure and enhance its photocatalytic activity. KNO nanostructures deliver significant H_2_ production under UV/visible irradiation with metal and non-metal doping further narrowing band gaps and enhancing visible light responsiveness.^12,13^ Its indirect band gap nature entails phonon-assisted electronic transitions, influencing optical absorption and recombination dynamics. Strategic band engineering enables strong photocatalytic potential for green hydrogen production from water splitting. Ongoing studies explore various doping strategies and first-principles investigations to improve its properties for real-world use. Pure KNO has a band gap of around 3.1 eV, absorbing mainly UV light. Its intrinsic photocatalytic hydrogen evolution under UV irradiation is moderate, but doping with metals such as Ni effectively tunes the band gap, in more specific Spin-up band gap values of K_1−*x*_Ni_*x*_NbO_3_: 0.956 eV (12.5% Ni), 0.957 eV (25% Ni), 1.040 eV (50% Ni), 0.876 eV (75% Ni).^14^ Co-doping or forming composites (*e.g.* CdS/Ni/KNbO_3_) further enhances light absorption and photocatalytic efficiency toward water splitting and pollutant degradation.^15^ Additionally, strategy combinations including oxygen vacancy engineering and co-catalyst addition improve not only hydrogen production but also other photocatalytic reactions such as nitrogen fixation. These modifications optimize charge carrier dynamics and increase the number of active sites, thereby expanding KNbO_3_ practical applicability.^16^

Density Functional Theory (DFT) studies confirm that these dopants and structural modifications enhance the electronic structure and improve photocatalytic stability. The increased surface area and doping also creates more active sites, boosting photocatalytic performance under visible light through tuning band gap to a moderate value.^17,18^ This study focuses on the effect of V doping on KNbO_3_ as a photocatalyst for water splitting using first-principles analysis mainly based on CASTEP module using two different functionals, GGA-PBE and mGGA-RSCAN, where the mGGA-RSCAN showed more accurate value than GGA-PBE, comparing with experimental values.^19–26^ Surface-level doping is critical, as catalytic activity primarily occurs at the material surface, where water molecule interactions and charge transfer take place. Previous studies have shown that V doping can increase the number of active sites and promote effective charge separation, thereby significantly improving the hydrogen evolution reaction efficiency.^27^

Using first-principles calculations based on DFT, our objective in the present work is to study the doped material at the quantum level to understand its electronic structure and the transitional behavior of the non-degenerate t_2g_ and e_g_ orbitals set of electrons for V compared to Nb, confirming through core level spectroscopy in the CASTEP module.^28^ To strengthen this study and get deeper understanding of V doping effect on the photocatalytic features of KNbO_3_ for water splitting, the water redox potential capability of undoped and V-doped KNbO_3_ photocatalysts are calculated.

## 2 Computational details

DFT calculations were performed using the Cambridge Serial Total Energy Package (CASTEP) module in Materials Studio 2020.^12^ Both Generalized Gradient Approximation-Perdew–Burke–Ernzerhof (GGA-PBE) and Meta-Generalized Gradient Approximation-Regularized Strongly Constrained and Appropriately Normed (mGGA-RSCAN) exchange–correlation functionals were employed to describe exchange–correlation interactions.^24–26^ Ultrasoft pseudopotentials (with PBE) and norm-conserving pseudopotentials (with RSCAN) were used for K, Nb, V, and O atoms. The Hubbard *U* correction (*U* = 5 eV) was applied to the Nb and V d orbitals to account for electron correlation.^27,28^ The KNbO_3_ structure (space group *Pm*3*m*) was modeled by cleaving with (1 0 0) surface plane and a 4 × 4 × 4 supercell to mitigate periodic boundary effects while the thickness of vacuum slab was 17 Å. A 600 eV(PBE) and 750 eV (RSCAN) plane-wave cutoff (PBE) ensured energy convergence^29^ while the surface layer of KNO chosen for substitutional doping and formed by KNb_1−*x*_V_*x*_O_3_ with *x* = 0.11. The Brillouin zone was sampled with a 6 × 6 × 6 Monkhorst–Pack grid. Structural optimization employed the LBFGS algorithm with convergence thresholds of 5 × 10^−6^ eV per atom (energy), 0.01 eV Å^−1^ (force), 0.02 GPa (stress), and 5 × 10^−4^ Å (displacement).^30,31^ Details about the computational parameters used in the calculations are reported in Table 1. Ionization energy and electron affinity were computed to determine Mulliken electronegativity for estimating conduction and valence band edge positions relative to the water redox potentials.^29^

**Table 1:** Computational details adopted for pristine and V-doped KNO using PBE and RSCAN functionals

Parameter	KNbO_3_ (GGA-PBE)	KNbO_3_ (mGGA-RSCAN)	KNb_0.89_V_0.11_O_3_ (GGA-PBE)	KNb_0.89_V_0.11_O_3_ (mGGA-RSCAN)
Pseudopotential type	Ultrasoft (PBE)	OTFG	Ultrasoft (PBE)	OTFG
		Norm-conserving (RSCAN)		Norm-conserving (RSCAN)
Hubbard *U* (Nb/V)	5 eV	5 eV	5 eV	5 eV
Supercell size	4 × 4 × 4	4 × 4 × 4	4 × 4 × 4	4 × 4 × 4
*k*-Point grid	6 × 6 × 6	6 × 6 × 6	6 × 6 × 6	6 × 6 × 6
Cutoff (eV)	600	750	600	750
Optimization algorithm	LBFGS	LBFGS	LBFGS	LBFGS
Energy convergence	<5 × 10^−6^ eV per atom	<5 × 10^−6^ eV per atom	<5 × 10^−6^ eV per atom	<5 × 10^−6^ eV per atom
Force convergence	<0.01 eV Å^−1^	<0.01 eV Å^−1^	<0.01 eV Å^−1^	<0.01 eV Å^−1^
Stress convergence	<0.02 GPa	<0.02 GPa	< 0.02 GPa	<0.02 GPa
Displacement convergence	<5 × 10^−4^ Å	<5 × 10^−4^ Å	<5 × 10^−4^ Å	<5 × 10^−4^ Å

## 3 Results and discussion

### 3.1 Structural properties

**Fig. 1:**
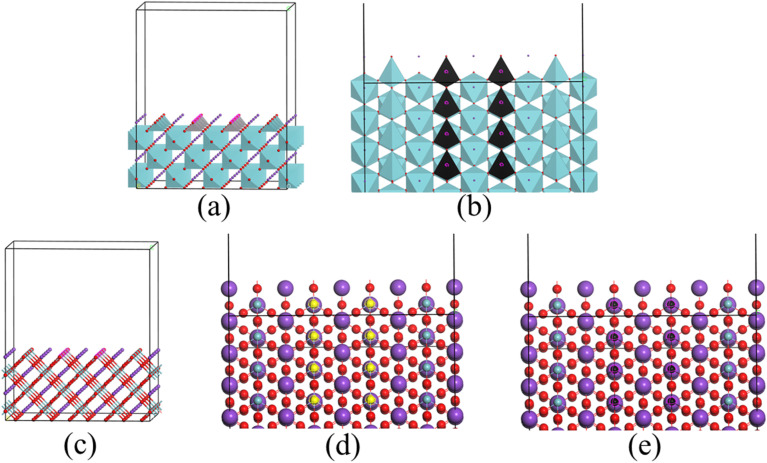
(a) Side view and (b) polyhedron top view of 11.11% V doped at the Nb sites in the surface (c) side view of slab generated from 4 × 4 × 4 supercell (d) top view of chosen Nb for substitution (yellow atoms) (e) top view of substituted V at the Nb site (black atoms).

The geometric optimization of both pure and V-doped KNbO_3_ was performed to achieve the lowest energy configuration and to understand the lattice distortions induced by V substitution.^32^ The cubic KNbO_3_ structure (space group *Pm*3*m*) includes five atoms per unit cell, with K at (0, 0, 0), Nb/V at (0.5, 0.5, 0.5), and O at face-centered positions (0, 0.5, 0.5). A 4 × 4 × 4 supercell minimizing periodic boundary effects during V substitution at Nb sites, exhibited excellent structural stability with lattice parameters close to experimental values.^33^ Post-optimization analysis revealed the structural ability of V doping, including altered Nb/V–O bond lengths and deviations in O–Nb/V–O bond angles from the ideal 180° configuration. A detailed comparison of the structural and energetic properties between pure and 11.11% V-doped on the surface of KNbO_3_ are shown in Table 2. It is shown that the lattice parameters obtained mGGA-RSCAN are the closest ones compared with the experimental values. Upon substituting 11.11% of Nb atoms with V at the surface sites, the total potential energy increased from 521.55 kcal mol^−1^ (pure) to 938.55 kcal mol^−1^ (doped), indicating a higher energetic state yet retaining stability.^13^ This energy rise correlates with increased bond and angle energies due to local strain fields arising from ionic radius mismatch between Nb^5+^ (0.64 Å) and V^5+^ (0.59 Å). Similarly, bond energy rises from 0.440453 to 6.560005 kcal mol^−1^ and angle energy increases from 517.8924 to 919.8595 kcal mol^−1^ (ref. 13 and 34) also becoming stronger with van der Waals interactions from 3.29937 to 12.2134 kcal mol^−1^.^13^ The total valence and non-bond energies show a marked increase as well, from 518.3328 to 926.4195 kcal mol^−1^ and from 3.218809 to 12.1293 kcal mol^−1^, respectively.^13,20,35^ The decrease in lattice constants (*a* = *b* = *c* = 3.943 Å for doped *versus* 4.019 Å for pure) and volume reduction (from 64.93 Å^3^ to 61.31 Å^3^) reflect lattice contraction, consistent with the replacement of a slightly smaller cation. Again, bond analysis showed shortened Nb/V–O bond lengths (average 1.96 Å in doped *versus* 2.008 Å in pure KNbO_3_), suggesting stronger metal–oxygen interactions and enhanced structural rigidity. This densification effect is also evident from increased calculated density (4.34 to 4.57 g cm^−3^), which are measured using mGGA-RSCAN,^12^ reflecting a more open lattice and indicating that the doped KNO can be a promising crystal for splitting water (Fig. 1).

**Table 2:** Structural and intermolecular information of pure and 11% V-doped KNO

Material	Total potential (kcal mol^−1^)	Bond energy (kcal mol^−1^)	Angle energy (kcal mol^−1^)	van der Waals energy (kcal mol^−1^)	*a* (Å)	*b* (Å)	*c* (Å)	Cell volume (Å^3^)	Density (g cm^−3^)	Functional	Reference
KNbO_3_	521.556	0.440	517.892	3.299	4.019	4.019	4.019	64.925	4.341	MGGA-RSCAN	This work
					4.091	4.091	4.091	68.417	4.575	GGA-PBE	This work
KNb_0.89_V_0.11_O_3_	938.549	6.560	919.860	12.213	3.943	3.943	3.943	61.312	4.570	MGGA-RSCAN	This work
					4.004	4.004	4.004	64.192	4.398	GGA-PBE	This work
KNbO_3_					4.022	4.022	4.022	65.061		Expt	36
					3.985	3.985	3.985	63.28		PBEsol	37
					3.989	3.989	3.989	63.47		AM05	37
					4.008	4.008	4.008	64.38		RTPSS	37
BaTiO_3_	—	—	—	—	3.996	3.996	4.036	64.281	6.020	GGA-PBEsol	38
SrTiO_3_	—	—	—	—	3.905	3.905	3.905	59.516	5.118	GGA-PBE	39
PbTiO_3_	—	—	—	—	3.879	3.879	4.161	62.600	7.980	GGA-PBEsol	38
NaTaO_3_	—	—	—	—	3.887	3.887	3.887	58.772	8.022	GGA-PBE	12
CaTiO_3_	—	—	—	—	3.806	5.380	5.438	111.231	3.984	GGA-PBE	40
LaAlO_3_	—	—	—	—	3.789	3.789	3.789	54.379	6.525	GGA-PBE	41

**Fig. 2:**
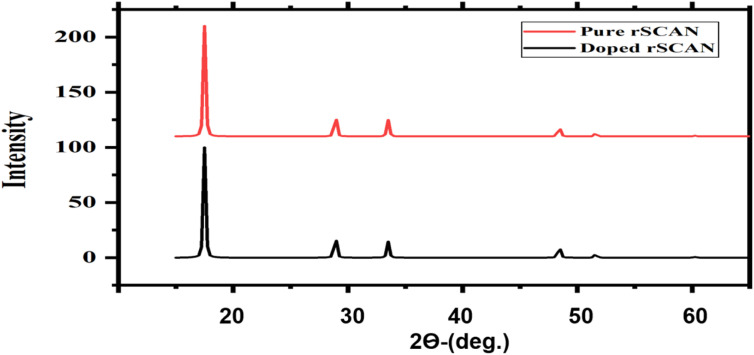
XRD analysis of cubic pure and V-doped KNO using DFT-RSCAN method.

These results are confirmed by analysis of the X-ray diffraction pattern in Fig. 2. Both “Pure” and “Dopped” samples display dominant peaks at similar 2-theta positions (around 18°, 29°, and 34°), characteristic of the cubic phase of KNbO_3_. The peak positions remain largely unchanged across the samples, indicating that the primary crystal symmetry (cubic) is preserved after doping. However, changes in intensity and the appearance of new small peaks in the doped sample suggest slight lattice distortions or defects, but do not indicate a phase transition. Slight shifts of peaks toward higher 2*θ* values indicate compressive strain, which may enhance electronic overlap between metal d and oxygen p orbitals, thereby benefiting photocatalytic activity.

### 3.2 Electronic properties

**Fig. 3:**
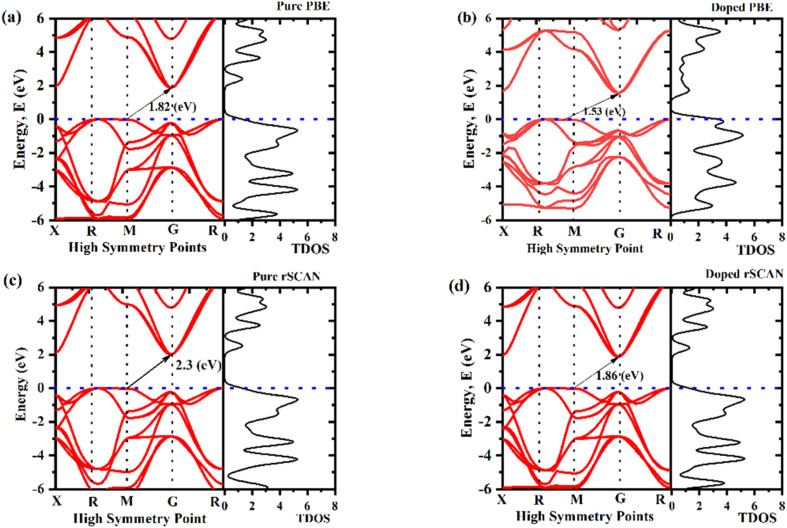
Calculated band structure and total density of states of (a) pure KNO using PBE (b) V-doped KNVO using PBE (c) pure KNO using RSCAN, and (d) V-doped KNO using RSCAN.

**Fig. 4:**
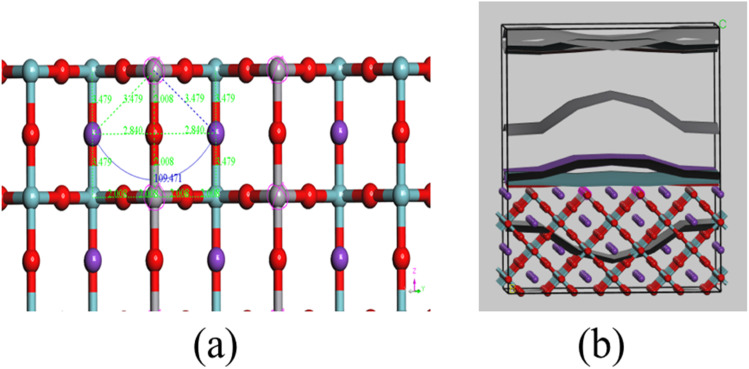
(a) Pre optimization bond length and angle distribution in V-doped KNO with K (in purple), Nb (in green), V (in gray), and O (in red). (b) Its corresponding density field.

The electronic band structure and density of states (DOS) analyses elucidate how V doping modulates the semiconducting behavior of KNbO_3_. For the pristine system, indirect band gaps of 1.82 eV (using PBE) and 2.30 eV (using RSCAN) were obtained in Fig. 3(a) and (c), respectively, consistent with previous theoretical studies (1.4–2.0 eV using GGA and up to 3.2 eV experimentally).^42^ According to a comprehensive DFT study using GGA (PBE, PBEsol) and *meta*-GGA (RTPSS) functionals, bandgap values for various KNbO_3_ structures ranged from 1.4 to 3.0 eV, depending on the functional,^37^ which is within the simulated band gap range. This wide range of band gap originates from the inherent limitations of different DFT functionals in describing electronic structure in GGA functional like PBE tend to underestimate the bandgaps due to the self-interaction error.^43–45^ The band structure of pure KNbO_3_ in Fig. 3(a) and (c) confirms that the valence and conduction bands are at different (M-G) points, hence proving it has an indirect band gap, while the non-zero value of TDOS near the Fermi level confirms KNO's semiconducting behavior.^29^ When a very small amount (11.11%) of intrinsic vanadium (V) is introduced in the niobium (Nb) site, it shows a reduced indirect band gaps of 1.53 eV and 1.86 eV, respectively, using PBE and RSCAN functionals, as shown in Fig. 3(d) and (b), which is much smaller than the pure KNbO_3_. The reduction in bandgap arises from the introduction of impurity states from V 3d orbitals that overlap with Nb 4d states in the conduction band, forming intermediate-energy levels near the Fermi level, as shown in Fig. 4(b).

**Fig. 5:**
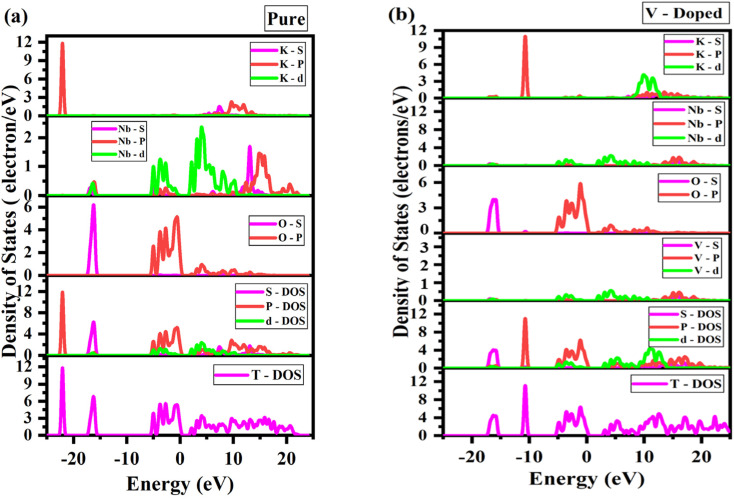
Calculated projected density states of (a) pure and (b) V-doped KNO using RSCAN.

These impurity bands act as intermediate states that effectively narrow the overall bandgap by allowing electrons to be excited by lower energy photons.^46^ This reduction in band gap improve the performance of photocatalytic water splitting by enhancing charge carrier separation as more electrons are scattering near Fermi level, thus leading to improved hydrogen generation compared with pure KNO.^47^Fig. 5(a) shows the density of states (DOS) of pure KNO in both its total and partial forms, where O 2p orbitals are the primary contributor of the VBM. Meanwhile, the CBM arises from the Nb 4d orbitals in pure KNbO_3_. When V is doped into KNO at 11.11%, the O 2p orbitals remain the primary contributor of the VB, whereas the predominant contribution of the CBM comes from a combination of V 3d and Nb 4d orbitals. That means the V 3d states are leading to the formation of CBM in doped KNO with minor contribution from Nb 4d states, as depicted in Fig. 5(b). This hybridization promotes charge transfer and enhances electron–hole separation. The presence of V 3d states within the forbidden gap introduces new electronic transitions, effectively extending absorption into the visible region.^13^

The charge density field maps further confirm the increased electron localization around V sites, demonstrating that V acts as an active center for photo-induced charge carrier dynamics. It seems that in Fig. 4(b), due to the V impurity, the electron density curve bends upward to the conduction band near the Fermi level, as it is a straight line in the case of pure KNbO_3_. That means when vanadium (V) partially (11.11%) substitutes for niobium (Nb) in the KNbO_3_ lattice surface electron density near the Fermi level increases. The presence of these intermediate bands facilitates more efficient excitation of electrons from the valence to conduction band. This rearranged electron density improves charge-carrier separation and mobility and reduces electron–hole recombination rates, which typically limit photocatalytic efficiency.^13^ This enhanced separation and increased availability of electrons near the Fermi level increase the rate of reduction reactions on the catalyst surface, thereby improving hydrogen evolution from water splitting.^13^

Comparatively, Ni-doped KNbO_3_ (bandgap 0.95–1.0 eV) and Fe-doped KNbO_3_ (1.5–1.7 eV)^46^ systems also show bandgap narrowing. In Table 3, the reduction in band gap of KNO using other dopant are shown as well However, excessive doping in these systems can lead to trap-assisted recombination. V doping maintains a balanced narrowing without creating deep trap states, indicating a better compromise between enhanced optical absorption and minimized recombination. Thus, moderate surface-level V incorporation optimizes the electronic structure for photocatalytic reactions.

**Table 3:** Calculated band gap values using different calculation methods

Material	Structure	Functional	Dopant	Band gap (eV)		Reference
				Pure	Dopped	
KNbO_3_	Cubic	GGA-PBE	Cr	2.14	1.78	48
	Cubic	GGA-PBE	Cr	2.14	1.78	48
	Cubic	GGA-PBE	Mn	2.14	1.67	48
	Cubic	GGA	Ti	2.9	2.2	35
	Cubic	GGA	Zn	2.92	2.11	35
	Cubic	HSE06	Ti	2.74	2.58	12
	Cubic	HSE06	V	2.74	1.08	12
	Tetragonal	GGA-PBE	—	1.35	—	49
	Cubic	GGA-PBE	V	1.82	1.53	This work
	Cubic	MGGA-rSCAN	V	2.30	1.86	This work

The density field and density of states (DOS) of a perovskite confirm^47^ that, when vanadium (V) partially substitutes for niobium (Nb) in the KNbO_3_ lattice (11.11%), it significantly alters the crystal electronic structure (Fig. 3(b) and (d)). This is because the electronic density rearranges due to the different valence electron configuration of vanadium, which has partially filled V 3d orbitals compared to Nb 4d orbitals.^47^Fig. 3(d) and (b) shows that the introduction of V atoms creates new energy levels within the original bandgap of pure KNbO_3_. Vanadium 3d impurity states form “stepping stones” near the Fermi level and bridge the gap between the valence and conduction bands. This effect is clearly visible in the DOS plots, where additional peaks appear within the forbidden energy region compared to pure KNbO_3_, indicating localized electronic states associated with the V dopant in Fig. 3(a–d).

### 3.3 Optical properties

#### 3.3.1 Dielectric function

The dielectric response is a key parameter governing the interaction of a photocatalyst with incident electromagnetic radiation and directly affects charge-carrier separation efficiency. A higher dielectric constant suppresses electron–hole recombination and improves optoelectronic as well as photocatalytic performance. The complex dielectric function is defined as:

1 *ε*(*ω*) = *ε*_1_(*ω*) + *iε*_2_(*ω*)

The real part *ε*_1_(*ω*), which represents the energy storage capability, can be obtained using the Kramers–Kronig relation:^50^2
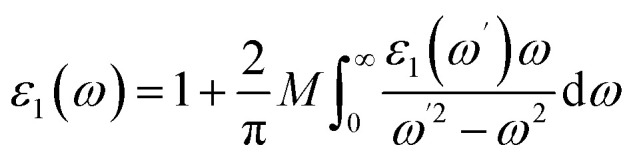


The imaginary part *ε*_2_(*ω*) is associated with interband transitions and energy dissipation, and can be calculated from the momentum matrix elements of occupied and unoccupied states:3
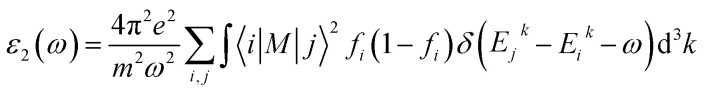


**Fig. 6:**
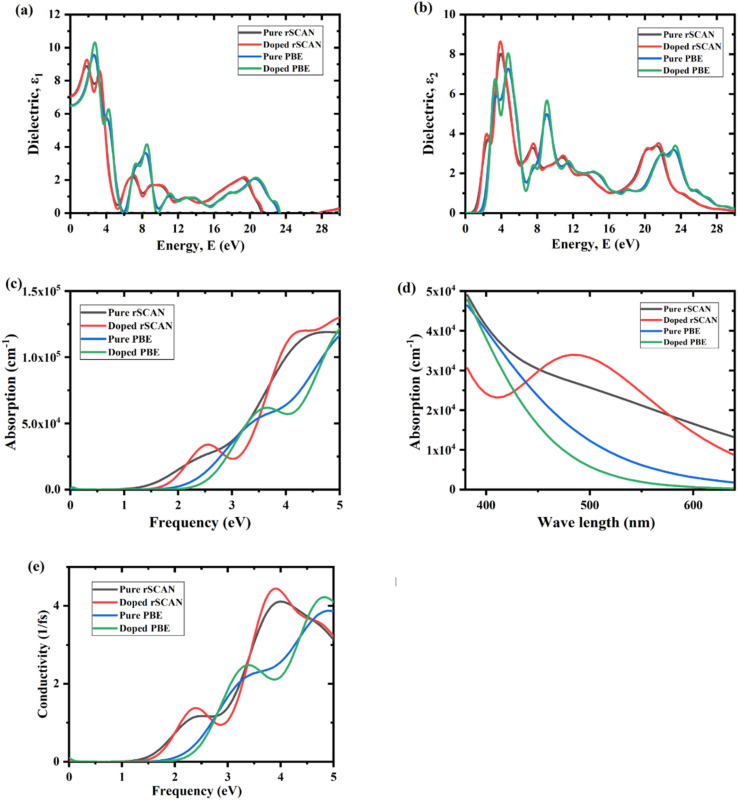
(a) Dielectric function (real), (b) dielectric function (img), (c) absorption *vs.* frequency, (d) absorption *vs.* nm (e) conductivity of pure and V-doped KNO using PBE and RSCAN.

Fig. 6(a and b) present the real and imaginary components of the dielectric function for pristine and V-doped KNbO_3_, calculated using PBE and RSCAN functionals. For the undoped system, the static dielectric constant is *ε*_1_(0) ≈ 6.5, whereas V substitution increases it up to ∼8.1 using RSCAN, indicating enhanced polarizability. The *ε*_1_(*ω*) spectra exhibit a pronounced peak below 4 eV, followed by a gradual decrease and oscillatory behavior at higher photon energies. At sufficiently high energies, *ε*_1_(*ω*) becomes negative, implying plasmonic resonance and metallic-like optical characteristics.

The *ε*_2_(*ω*) spectra show dominant absorption peaks in the 2–6 eV range, primarily due to O 2p → Nb/V 3d interband transitions. Doping increases peak intensities and induces slight spectral shifts, showing stronger enhancement using RSCAN than PBE. Beyond 6 eV, weaker transitions gradually diminish above 14 eV. Although both functionals exhibit similar qualitative behavior, the quantitative differences underscore the sensitivity of the dielectric response to the exchange–correlation treatment. These results confirm that V doping significantly strengthens polarization effects, enhances low-energy optical activity, and improves transition probabilities relevant to photocatalytic performance.

#### 3.3.2 Absorption

Fig. 6(c and d) illustrates the absorption behavior of the material in both pure and doped configurations, evaluated using the RSCAN and PBE exchange–correlation functionals, offering a comprehensive view of the optical response across the visible and ultraviolet regions. In Fig. 6(d), where the absorption coefficient is plotted *versus* wavelength, all samples exhibit a gradual decline in absorption intensity with increasing wavelength, which is typical for wide-band-gap semiconductors where absorption diminishes as photon energy decreases below interband transition thresholds.^51^ The RSCAN functional consistently predicts higher absorption values compared to PBE, reflecting its enhanced capability in capturing interband transition strength and providing superior treatment of electronic correlations in transition metal oxide systems.^52^ The doped systems exhibit clear modifications in the optical response: for RSCAN, a prominent absorption enhancement appears around ∼480–520 nm, attributed to defect-induced intermediate states that create additional transition pathways within the band gap.^53^ Similarly, doping under PBE slightly elevates absorption in the mid-visible range, although less prominently than RSCAN, consistent with the typical underestimation of transition strength in semi-local functionals.^12^ These observations confirm that introducing dopants modulates optical transitions and improves visible-light harvesting, as reported in transition-metal doped perovskite photocatalysts.^13^ The photon-energy-dependent absorption spectra in Fig. 6(c) further emphasize this trend, where the absorption coefficient increases steadily with photon energy for all cases, consistent with strong interband transitions arising beyond the fundamental bandgap.^54^ Notably, the doped RSCAN system exhibits the highest absorption intensity within 3–4.5 eV, indicating that doping substantially enhances optical excitation probabilities when evaluated using a more accurate functional, while pure and doped PBE show parallel trends with comparatively lower magnitudes.^55^ Overall, the results clearly indicate that both doping and exchange–correlation functional choice play significant roles in determining the optical absorption characteristics, with the SCAN functional exhibiting higher sensitivity to structural and electronic perturbations, predicting stronger and more tunable absorption behavior, an advantageous attribute for photovoltaic and optoelectronic applications.^56^

#### 3.3.3 Conductivity

The frequency-dependent optical conductivity *σ*(*ω*) of pure and V doped KNbO_3_, computed using both PBE and RSCAN exchange–correlation functionals, reveals important insights into how dopants enhance light absorption. As shown in Fig. 6(e), all configurations display negligible conductivity below ∼1.5 eV, indicating an absence of low-energy interband transitions, which is consistent with established optical response mechanisms in perovskite niobates where optical conductivity remains suppressed below the fundamental band gap energy prior to sharp elevation due to interband absorption.^57^ Beyond this energy threshold, the conductivity increases progressively, coinciding with the onset of significant photon-induced electronic transitions. The impact of vanadium doping becomes strikingly apparent when comparing spectra: the pure RSCAN calculation shows a gradual conductivity increase with a maximum at approximately 4.0 eV, whereas the doped RSCAN sample exhibits substantially enhanced conductivity across the entire spectrum, particularly between 3.0 and 4.5 eV, demonstrating that dopant incorporation effectively promotes carrier activation and generates new transition pathways, an enhancement that aligns with reported observations in transition-metal doped KNbO_3_ systems.^12^ The PBE functional reveals analogous behavior, with the doped structure displaying a more intense and sharper conductivity peak (∼4.2 eV) compared to the comparatively moderate response of the pure PBE system. Importantly, the conductivity enhancement from doping is consistent across both computational methods, originating from dopant-induced electronic states that simultaneously increase absorption coefficients and modulate band curvature, as observed in metal-doped perovskite photocatalysts.^13^ The differences between PBE and RSCAN predictions are informative, as RSCAN provides better treatment of electronic correlations in transition metal oxides,^58^ while both methods consistently demonstrate enhanced conductivity in doped structures. These findings establish V-doped KNbO_3_ as a promising material for photovoltaic and optoelectronic applications requiring strong light–matter interactions.^56^

### 3.4 Redox properties

To understand the water-splitting capability of our material, there are various ways to determine it; one is to compare the valence band maximum (VBM) and conduction band minimum (CBM) energy positions with respect to water redox potentials.^29^ The process of water splitting is divided into two main parts. First, the photon is absorbed by the semiconductive material from sunlight, and the water is split into H^+^ and OH^−^. Then, the OH^−^ oxidizes at the valence band of the photocatalyst and forms oxygen (O_2_), also known as oxygen evolution reaction (OER), while the H^+^ reduces at the conduction band to form Hydrogen (H_2_), also known as hydrogen evolution reaction (HER). This is the process by which we generate hydrogen at the catalyst surface. Reduction and oxidation reactions, respectively, can be triggered by the photogenerated electrons and holes.

In order to achieve a suitable water splitting process, the VBM energy position of must lie below the oxidation potential of H_2_O to O_2_ (1.23 V at pH = 0 *vs.* normal hydrogen electrode, NHE), while the CBM energy position must lie above the reduction potential of H^+^ to H_2_ (0 V *vs.* NHE).^59^

The VBM and CBM energy positions can be determined through following formula:

4 *E*_CBM_ = *χ* − *E*_e_ − ½*E*_g_

**Fig. 7:**
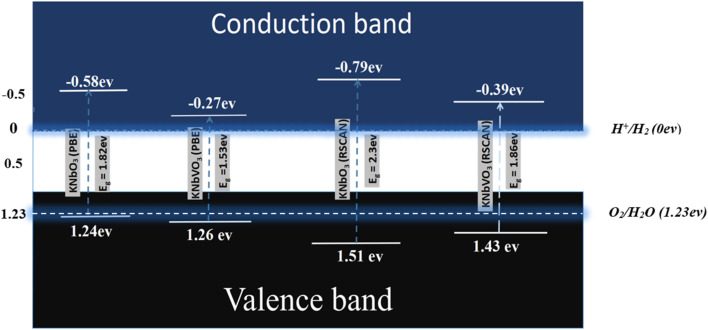
VBM and CBM energy edges with respect to water redox potential of pure and V-doped KNO using PBE and RSCAN.

5 *E*_VBM_ = *E*_CBM_ + *E*_g_

where *E*_g_ is the band gap energy of the material, *E*_CBM_ is the CBM energy position, and *E*_VBM_ is the VBM energy position. *χ* is the Mulliken electronegativity, which is mainly the average of electron affinity and 1st ionization energy of the material, and *E*_e_ is the energy of free electrons on the NHE scale (−4.5 eV). Using Mulliken electronegativity calculations, KNbO_3_ exhibits *E*_CBM_ of −0.58 eV (using PBE) and −0.79 eV (using RSCAN), while V-doped KNbO_3_ shows *E*_CBM_ of −0.27 eV (using PBE) and −0.39 eV (using RSCAN), as shown in Fig. 7.

All CBM energy values are above the H^+^/H_2_ redox potential (0 eV), enabling hydrogen evolution reaction. Although the effect of Vanadium reduces the reduction strength of the material by showing less negative CBM energy value than the pristine, V-doped KNbO_3_ maintains its ability for hydrogen production. Similarly, all VBM energy positions are below 1.23 eV, satisfying the O_2_/H_2_O oxidation potential requirement. Thus, both pure and V-doped KNbO3 are thermodynamically capable of splitting water into H_2_ and O_2_. Moreover, this reduction in electronic band gap upon V doping is expected to improve the optical properties and overcome this limitation in KNbO_3_ photocatalyst for being used in visible-light-driven hydrogen production (Table 4).

**Table 4:** Mulliken electronegativity and edge potential of pure and doped KNO using PBE and RSCAN

Material	Functional	*E*_a_ (eV) (electron affinity)	*I*_i_ (eV) (ionization potential)	*χ* (eV) (Mulliken electronegativity)	*E*_CB_ (eV) (conduction band)	*E*_VB_ (eV) (valence band)	DOI/reference
KNbO_3_	GGA-PBE	1.47	8.19	4.83	−0.58	1.24	This work
KNbVO_3_ (11% V doped)	GGA-PBE	1.67	8.32	4.995	−0.27	1.26	This work
KNbO_3_	mGGA-RSCAN	1.47	8.24	4.86	−0.79	1.51	This work
KNbVO_3_ (11% V doped)	mGGA-RSCAN	1.49	8.39	5.02	−0.39	1.43	This work
BaTiO_3_	GGA-PBE	—	—	5.33	−0.67	2.53	60
SrTiO_3_	GGA-PBE	—	—	5.13	−0.63	2.62	60
PbTiO_3_	GGA-PBE	—	—	5.19	−0.59	2.81	61
NaTaO_3_	GGA-PBE	—	—	5.55	−1.00	3.00	62
CaTiO_3_	HSE06	—	—	5.47	−0.73	2.57	40
LaAlO_3_	GGA-PBE	—	—	5.22	−0.72	2.98	61

The moderate downward shift in the CBM energy position with V doping enhances electron transfer to adsorbed protons, facilitating hydrogen evolution reaction. Improved dielectric screening and reduced electron–hole recombination raise quantum efficiency. Compared to other transition-metal-doped perovskites, V-doped KNbO_3_ offers better stability and electronic configuration due to V optimal d-electron occupation (3d^3^4s^2^), which promotes delocalized conduction band states. Lattice strain, bandgap narrowing, and ideal band edge alignment together make KNb_1−*x*_V_*x*_O_3_ a promising photocatalyst for green hydrogen production.

## 4 Conclusion

In this work, DFT calculations using the two PBE and RSCAN exchange–correlation functionals were carried out to understand the water-splitting capability of both pure and V-doped KNbO_3_ perovskite, which is well known for its higher efficiency in solar energy conversion. Our calculations demonstrated that V-doped KNbO_3_ exhibits better electronic and optical properties for photocatalytic water splitting as compared to undoped KNbO_3_. Vanadium incorporation narrowed the bandgap, enhanced visible-light absorption, and improved electronic conductivity. To be more specific, a band gap reduction from 1.82 eV to 1.53 eV (using PBE) and from 2.30 eV to 1.86 eV (using RSCAN) was achieved through an 11.11% surface doping of V at the Nb sites. An X-ray diffraction analysis demonstrated that this material has confirmed its cubic phase crystalline structure. The calculated VBM and CBM positions met the thermodynamic criteria for water splitting, confirming the photocatalytic feasibility.

Further studies can be conducted, such as adsorbing hydrogen and water molecules onto the materials and calculating their Gibbs free energies, to better understand their water-splitting capability. The RSCAN functional provided results closer to experimental observations than PBE, highlighting its importance for giving better accuracy in modelling perovskite photocatalysts.

The findings of our study shed light on the significance of V-doped KNbO_3_ for achieving efficient hydrogen generation *via* photocatalytic water splitting. This presents a promising avenue for developing green hydrogen generation and sustainable energy solutions.

## Author contributions

Aktab Quadir Sreshtho: simulation analysis, conceptualization, investigation, writing – original draft, writing – review & editing, formal analysis; Sourav Chandra Das: investigation, writing – original draft, writing – review & editing; Tanvir Aftab Talal: simulation analysis, plotting graph; Md Hasnain: validation, writing – review & editing, formal analysis; Joy Biswas: formal analysis; Akdas Quadir Shwapno: formal analysis; Riddi Barua: formal analysis; Moussab Harb: conceptualization, writing – review & editing, validation, supervision.

## Conflicts of interest

The authors declare no competing interests.

## Data Availability

The datasets generated and analyzed during the current study are available from the corresponding author on reasonable request. The structural input files, CASTEP parameter settings, and processed data supporting the findings of this work can be provided upon request for academic research purposes.

## References

[cit1] Lima M. A. F. B., Carvalho P. C. M., Fernández-Ramírez L. M., Braga A. P. S. (2020). Improving Solar Forecasting Using Deep Learning and Portfolio Theory Integration. Energy.

[cit2] Singh V., Singh R., Arora K. S., Mahajan D. K. (2019). Hydrogen Induced Blister Cracking and Mechanical Failure in X65 Pipeline Steels. Int. J. Hydrogen Energy.

[cit3] Yin X., Zhang W., Jiang Z., Pan L. (2020). Data-Driven Multi-Objective Predictive Control of Offshore Wind Farm Based on Evolutionary Optimization. Renewable Energy.

[cit4] Babaa S., Khzouz M. (2024). Systems Engineering Department, Military Technological College (Affiliated with University of Portsmouth, UK), Muscat, Oman. Hydrogen Production Methods: A Literature Review. Adv. Image Video Process..

[cit5] Wang B., Yu H., Wang M., Han L., Wang J., Bao W., Chang L. (2021). Microwave Synthesis Conditions Dependent Catalytic Performance of Hydrothermally Aged CuII-SSZ-13 for NH3-SCR of NO. Catal. Today.

[cit6] Li S., Wen Z., Hou J., Xi S., Fang P., Guo X., Li Y., Wang Z., Li S. (2022). Effects of Ethanol and Methanol on the Combustion Characteristics of Gasoline with the Revised Variation Disturbance Method. ACS Omega.

[cit7] Tournet J., Lee Y., Karuturi S. K., Tan H. H., Jagadish C. (2020). III–V Semiconductor Materials for Solar Hydrogen Production: Status and Prospects. ACS Energy Lett..

[cit8] Kato H., Asakura K., Kudo A. (2003). Highly Efficient Water Splitting into H_2_ and O_2_ over Lanthanum-Doped NaTaO_3_ Photocatalysts with High Crystallinity and Surface Nanostructure. J. Am. Chem. Soc..

[cit9] Ding Q.-P., Yuan Y.-P., Xiong X., Li R.-P., Huang H.-B., Li Z.-S., Yu T., Zou Z.-G., Yang S.-G. (2008). Enhanced Photocatalytic Water Splitting Properties of KNbO_3_ Nanowires Synthesized through Hydrothermal Method. J. Phys. Chem. C.

[cit10] Yan L., Zhang J., Zhou X., Wu X., Lan J., Wang Y., Liu G., Yu J., Zhi L. (2013). Crystalline Phase-Dependent Photocatalytic Water Splitting for Hydrogen Generation on KNbO3 Submicro-Crystals. Int. J. Hydrogen Energy.

[cit11] Zhang T., Zhao K., Yu J., Jin J., Qi Y., Li H., Hou X., Liu G. (2013). Photocatalytic Water Splitting for Hydrogen Generation on Cubic, Orthorhombic, and Tetragonal KNbO3 Microcubes. Nanoscale.

[cit12] Liang Y., Shao G. (2019). First Principles Study for Band Engineering of KNbO_3_ with 3d Transition Metal Substitution. RSC Adv..

[cit13] Maarouf A. A., Gogova D., Fadlallah M. M. (2021). Metal-Doped KNbO3 for Visible Light Photocatalytic Water Splitting: A First Principles Investigation. Appl. Phys. Lett..

[cit14] Rafique F., Ishfaq M., Aldaghfag S. A., Yaseen M., Zahid M., Butt M. K. (2023). First Principles Insight into Magnetic and Optoelectronic Properties of Ni Doped KNbO3 Perovskite. J. Ovonic Res..

[cit15] Ryu S. Y., Lee T. K., Hoffmann M. R. (2023). Spectroscopic Study on CdS/Ni/KNbO_3_ : Confirming Ni Effect to Photocatalytic Activity. ACS Omega.

[cit16] Xia C., Teng J., Yue L., Zheng Y., Chu Y., Zhuang L., Zhao L., He Y. (2025). Enhanced Photocatalytic Nitrogen Fixation over MnO *x* -Modified O-KNbO3 : Impact of Oxygen Vacancies and MnO *x* Loading. J. Mater. Chem. C.

[cit17] Valadares F., Guilhon I., Teles L. K., Marques M. (2020). Atomistic Origins of Enhanced Band Gap, Miscibility, and Oxidation Resistance in α-CsPb 1– *x* Sn *x* I3 Mixed Perovskite. J. Phys. Chem. C.

[cit18] Wang W., Zhou W., Li W., Xiong X., Wang Y., Cheng K., Kang J., Zhang Q., Wang Y. (2020). In-Situ Confinement of Ultrasmall Palladium Nanoparticles in Silicalite-1 for Methane Combustion with Excellent Activity and Hydrothermal Stability. Appl. Catal., B.

[cit19] Bantawal H., Shenoy U. S., Bhat D. K. (2021). Vanadium Doped CaTiO3 Cuboids: Role of Vanadium in Improving the Photocatalytic Activity. Nanoscale Adv..

[cit20] Farooq U., Chaudhary P., Ingole P. P., Kalam A., Ahmad T. (2020). Development of Cuboidal KNbO3 @α-Fe2 O3 Hybrid Nanostructures for Improved Photocatalytic and Photoelectrocatalytic Applications. ACS Omega.

[cit21] Faghihnasiri M., Izadifard M., Ghazi M. E. (2017). DFT Study of Mechanical Properties and Stability of Cubic Methylammonium Lead Halide Perovskites (CH3 NH3 PbX3 , X = I, Br, Cl). J. Phys. Chem. C.

[cit22] Al-Zahrani H. Y. S., El Radaf I. M., Lahmar A. (2025). A Study on the Impact of Vanadium Doping on the Structural, Optical, and Optoelectrical Properties of ZnS Thin Films for Optoelectronic Applications. Micromachines.

[cit23] Santra G., Martin J. M. L. (2021). Pure and Hybrid SCAN, rSCAN, and r2SCAN: Which One Is Preferred in KS- and HF-DFT Calculations, and How Does D4 Dispersion Correction Affect This Ranking?. Molecules.

[cit24] Yates J. R., Bartók A. P. (2025). Accurate Predictions of Chemical Shifts with the rSCAN and r2 SCAN mGGA Exchange–Correlation Functionals. Faraday Discuss..

[cit25] Kothakonda M., Kaplan A. D., Isaacs E. B., Bartel C. J., Furness J. W., Ning J., Wolverton C., Perdew J. P., Sun J. (2023). Testing the r2 SCAN Density Functional for the Thermodynamic Stability of Solids with and without a van Der Waals Correction. ACS Mater. Au.

[cit26] Doumont J., Tran F., Blaha P. (2022). Implementation of Self-Consistent MGGA Functionals in Augmented Plane Wave Based Methods. Phys. Rev. B.

[cit27] Li Y., Li L., Liu F., Wang B., Gao F., Liu C., Fang J., Huang F., Lin Z., Wang M. (2022). Robust Route to H2O2 and H2 via Intermediate Water Splitting Enabled by Capitalizing on Minimum Vanadium-Doped Piezocatalysts. Nano Res..

[cit28] Gao S.-P., Pickard C. J., Perlov A., Milman V. (2009). Core-Level Spectroscopy Calculation and the Plane Wave Pseudopotential Method. J. Phys.: Condens. Matter.

[cit29] Hossian Md. S., Babu Md. M. H., Kabir A., Azzouz Rached A., Kholil Md. I. (2025). A DFT Insight into Lead Free Double Halide Perovskite Cs2 TeI6 for Clean and Renewable Energy Sources. Mater. Adv..

[cit30] Hasan N., Arifuzzaman M., Kabir A. (2022). Structural, Elastic and Optoelectronic Properties of Inorganic Cubic FrBX3 (B = Ge, Sn; X = Cl, Br, I) Perovskite: The Density Functional Theory Approach. RSC Adv..

[cit31] Erum N., Ahmad J. (2023). Structural, Elastic and Mechanical Properties of Cubic Perovskite Materials. Arch. Adv. Eng. Sci..

[cit32] Chen H.-L., Ju S.-P., Lin C.-Y., Pan C.-T. (2018). Investigation of Microstructure and Mechanical Properties of Polyvinylidene Fluoride/Carbon Nanotube Composites after Electric Field Polarization: A Molecular Dynamics Study. Comput. Mater. Sci..

[cit33] Materials Data on KNbO3 by Materials Project, 2020, 10.17188/1313155

[cit34] Zhang X., Qi R., Dong S., Yang S., Jing C., Sun L., Chen Y., Hong X., Yang P., Yue F., Chu J. (2021). Modulation of Ferroelectric and Optical Properties of La/Co-Doped KNbO3 Ceramics. Nanomaterials.

[cit35] Wang D., Wang G., Lu Z., Al-Jlaihawi Z., Feteira A. (2020). Crystal Structure, Phase Transitions and Photoferroelectric Properties of KNbO3-Based Lead-Free Ferroelectric Ceramics: A Brief Review. Front. Mater..

[cit36] Fontana M. D., Metrat G., Servoin J. L., Gervais F. (1984). Infrared Spectroscopy in KNbO3 through the Successive Ferroelectric Phase Transitions. J. Phys. C: Solid State Phys..

[cit37] Schmidt F., Landmann M., Rauls E., Argiolas N., Sanna S., Schmidt W. G., Schindlmayr A. (2017). Consistent Atomic Geometries and Electronic Structure of Five Phases of Potassium Niobate from Density-Functional Theory. Adv. Mater. Sci. Eng..

[cit38] Balachandran P. V., Xue D., Lookman T. (2016). Structure–Curie Temperature Relationships in BaTiO 3 -Based Ferroelectric Perovskites: Anomalous Behavior of ( Ba , Cd ) TiO 3 from DFT, Statistical Inference, and Experiments. Phys. Rev. B.

[cit39] Yuk S. F., Pitike K. C., Nakhmanson S. M., Eisenbach M., Li Y. W., Cooper V. R. (2017). Towards an Accurate Description of Perovskite Ferroelectrics: Exchange and Correlation Effects. Sci. Rep..

[cit40] Jouybar S., Naji L., Tafreshi S. S., Mozaffari S. A., De Leeuw N. H. (2024). Density Functional Theory Study of Alkaline Earth-Based Titanate Perovskite Oxides: Unraveling Their Significance for Solar Cell Applications. J. Phys. Chem. C.

[cit41] El-Mellouhi F., Brothers E. N., Lucero M. J., Bulik I. W., Scuseria G. E. (2013). Structural Phase Transitions of the Metal Oxide Perovskites SrTiO 3 , LaAlO 3 , and LaTiO 3 Studied with a Screened Hybrid Functional. Phys. Rev. B: Condens. Matter Mater. Phys..

[cit42] Ding Q.-P., Yuan Y.-P., Xiong X., Li R.-P., Huang H.-B., Li Z.-S., Yu T., Zou Z.-G., Yang S.-G. (2008). Enhanced Photocatalytic Water Splitting Properties of KNbO3 Nanowires Synthesized through Hydrothermal Method. J. Phys. Chem. C.

[cit43] Tran F., Blaha P. (2009). Accurate Band Gaps of Semiconductors and Insulators with a Semilocal Exchange-Correlation Potential. Phys. Rev. Lett..

[cit44] Abbas A. T. Y. , Alfahed R. K. F. and Badran H. A., Application the Synchronization Method of Adaptive-Observer, Al-Samawa, Iraq, 2021, p 070001, 10.1063/5.0068906

[cit45] Li R., Luan J., Zhang Y., Jiang L., Yan H., Chi Q., Yan Z. (2024). A Review of Efficient Photocatalytic Water Splitting for Hydrogen Production. Renewable Sustainable Energy Rev..

[cit46] Apostolova I. N., Apostolov A. T., Wesselinowa J. M. (2024). Band Gap and Polarization Tuning of Ion-Doped XNbO3 (X = Li, K, Na, Ag) for Photovoltaic and Energy Storage Applications. Molecules.

[cit47] Endres J., Egger D. A., Kulbak M., Kerner R. A., Zhao L., Silver S. H., Hodes G., Rand B. P., Cahen D., Kronik L., Kahn A. (2016). Valence and Conduction Band Densities of States of Metal Halide Perovskites: A Combined Experimental–Theoretical Study. J. Phys. Chem. Lett..

[cit48] Steven P. L. , William M. D. and E. G. S., Ab Initio Studies of Dopant-Defect Complexes in KNbO3, 2014. https://openscholar.uga.edu/record/4134/files/suter_eric_g_202203.pdf

[cit49] Monira M., Helal M. A., Liton M. N. H., Kamruzzaman M., Kojima S. (2023). Elastic, Optoelectronic and Photocatalytic Properties of Semiconducting CsNbO3: First Principles Insights. Sci. Rep..

[cit50] Liu X., Xie B., Duan C., Wang Z., Fan B., Zhang K., Lin B., Colberts F. J. M., Ma W., Janssen R. A. J., Huang F., Cao Y. (2018). A High Dielectric Constant Non-Fullerene Acceptor for Efficient Bulk-Heterojunction Organic Solar Cells. J. Mater. Chem. A.

[cit51] Banerjee S., Pillai S. C., Falaras P., O'Shea K. E., Byrne J. A., Dionysiou D. D. (2014). New Insights into the Mechanism of Visible Light Photocatalysis. J. Phys. Chem. Lett..

[cit52] Furness J. W., Kaplan A. D., Ning J., Perdew J. P., Sun J. (2020). Accurate and Numerically Efficient r2 SCAN Meta-Generalized Gradient Approximation. J. Phys. Chem. Lett..

[cit53] Khan A., Chatterjee S., Nath T. K., Taraphder A. (2021). Defect-Induced Modulation of Magnetic, Electronic, and Optical Properties of the Double-Perovskite Oxide La 2 CoMnO 6. Phys. Rev. B.

[cit54] Frati F., Hunault M. O. J. Y., De Groot F. M. F. (2020). Oxygen K-Edge X-Ray Absorption Spectra. Chem. Rev..

[cit55] Hasan M. R., Apon I. A., Islam M. M., Azad A. U., Solayman M., Haque M. S. (2025). Pressure-Induced Multi-Functional Property Analysis of Lead-Free Tin Based Halide Perovskites ASnCl3 (A = Ga, In, Tl) for Advanced Optoelectronic Applications. Mater. Adv..

[cit56] Hasan Z., Rahman M. A., Das D. K., Rouf H. K. (2023). Influence of Ca Doping in Structural, Electronic, Optical and Mechanical Properties of Ba1−xCaxTiO3 Perovskite from First-Principles Investigation. Sci. Rep..

[cit57] Varrassi L., Liu P., Yavas Z. E., Bokdam M., Kresse G., Franchini C. (2021). Optical and Excitonic Properties of Transition Metal Oxide Perovskites by the Bethe-Salpeter Equation. Phys. Rev. Mater..

[cit58] Saikot M. S. H., Rafiu R., Apon I. A., El-Rayyes A., Rahman M. A., Shkir M., Ahmad Z., Marnadu R. (2025). First-Principles Investigation of Structural, Electronic, Optical, Mechanical, and Phonon Properties of Pb- and Sn-Based Cubic Oxide Perovskites for Optoelectronic Applications. RSC Adv..

[cit59] Chen X., Shen S., Guo L., Mao S. S. (2010). Semiconductor-Based Photocatalytic Hydrogen Generation. Chem. Rev..

[cit60] Jacobs R., Booske J., Morgan D. (2016). Understanding and Controlling the Work Function of Perovskite Oxides Using Density Functional Theory. Adv. Funct. Mater..

[cit61] Li W., Wang Z., Xiao X., Zhang Z., Janotti A., Rajasekaran S., Medasani B. (2022). Predicting Band Gaps and Band-Edge Positions of Oxide Perovskites Using Density Functional Theory and Machine Learning. Phys. Rev. B.

[cit62] Singh A. P., Kumar S., Thirumal M. (2019). Efficient Charge Transfer in Heterostructures of CdS/NaTaO3 with Improved Visible-Light-Driven Photocatalytic Activity. ACS Omega.

